# The association between Internet use and cognitive ability among rural left-behind children in China

**DOI:** 10.3389/fpubh.2023.1341298

**Published:** 2024-01-22

**Authors:** Ai-zhi Gao, Wei-chao Chen

**Affiliations:** Hunan Normal University, Changsha, China

**Keywords:** left-behind children, Internet use, cognitive ability, instrumental variable (IV) approach, parental migration

## Abstract

**Introduction:**

This study focuses on the cognitive development of rural children aged 10–15 who have been left behind, utilizing data from the China Family Panel Studies (CFPS) datasets of 2016 and 2020. The primary objective is to investigate the correlation between Internet usage and the cognitive ability of these children.

**Methods:**

An Ordinary Least Squares (OLS) regression model was initially employed to explore the potential influence of Internet use on the cognitive ability of rural left-behind children. To meticulously address potential endogeneity, we employed the instrumental variable (IV) method. Additionally, we performed robustness checks using Propensity Score Matching (PSM) to ensure the reliability of our findings.

**Results:**

The findings indicate a statistically significant positive correlation between Internet usage and the cognitive ability of left-behind rural children. Notably, the impact of Internet use is more pronounced in girls than in boys among this demographic. Furthermore, a significant influence of Internet usage on the cognitive ability is observed in rural children aged 10–12, whereas no significant correlation is found for those aged 13–15. Particularly noteworthy is the substantial impact of Internet use on the cognitive ability of left-behind children with an absent father. In addition, the cognitive benefits associated with Internet use were notably more pronounced among rural left-behind children, especially when considering factors such as attendance at a demonstration school and parental concern for the child’s education.

**Conclusion:**

This study underscores the importance of understanding the relationship between Internet usage and cognitive development in left-behind rural children. These findings highlight the need for targeted interventions and inclusive access to online resources for the development of rural left-behind children.

## Introduction

The process of urbanization has precipitated an influx of rural laborers into urban areas, culminating in a substantial rise in the population of rural left-behind children in China. The 2021 data from the Chinese Ministry of Civil Affairs reveals that as of the end of the 13th Five-Year Plan period, there were a total of 6.436 million rural left-behind children in China (1). Adolescence is a pivotal stage in cognitive and psychological development. Research underscores the pivotal role of parental companionship in influencing both cognitive and non-cognitive development among junior high school-aged children (2). Notably, the act of being left behind bears a significant negative impact on children’s cognitive ability (3).

The Internet has undergone a transformative journey, evolving from a simple source of entertainment to a versatile tool deeply ingrained in both production and daily life. Its widespread usage has rendered it indispensable in households, where it serves myriad purposes such as online learning, entertainment, and remote communication. This shift positions the Internet as a viable alternative to address educational gaps, particularly for rural left-behind children. A significant turning point occurred in July 2021 when the Chinese Government introduced the ‘Double-Reduction’ policy, sparking widespread discussion. This initiative sought to alleviate the academic burden on students by reducing homework while simultaneously tightening regulations on private tutorial companies. The outcome of this policy is a surplus of time for students after school, potentially leading to increased access to the Internet. After-school programs, crafted to augment students’ academic performance, have demonstrated a significant impact on their cognitive development. Gardner (4) posited that after-school programs, specifically crafted to improve students’ academic performance, exerted a notable influence on their cognitive development. Notably, individuals who adeptly utilized computers within these programs showcased superior performance in vocabulary, reading comprehension, and numeracy, surpassing the mean scores reported by Attewell et al. (5). This correlation underscores the potential benefits of strategic Internet use in after-school settings, suggesting a positive impact on diverse cognitive skills.

In this context, the additional free time can be viewed as an opportunity for students to engage with online resources, educational content, and a plethora of learning materials available on the Internet. The shift toward increased Internet usage aligns with the changing landscape of education, providing students with a platform to explore and enhance their knowledge beyond traditional classroom settings. The intersection of reduced academic pressure and heightened Internet access could pave the way for a more dynamic and personalized learning experience for students. As they navigate the online landscape, they can explore diverse educational avenues and capitalize on the wealth of information at their fingertips.

Despite an increasing body of research on the Internet’s crucial role in the cognitive development of adolescents, a consensus remains elusive. Scholars are divided, with some contending that the Internet reshapes cognitive processes through digital communication, significantly enhancing adolescents’ reading comprehension and logical deduction abilities (6). Conversely, an opposing viewpoint posits that the Internet’s features, such as online learning and communication, hinder the personality development of adolescents, potentially leading to Internet addiction and subsequent academic impediments (7).

While studies on the relationship between Internet use and the cognitive ability of children abound, there is a dearth of research specifically exploring the impact on rural left-behind children. As a pervasive medium, the Internet may function as an alternative educational tool to compensate for the absence of familial and school-based education for these children. Additionally, an in-depth examination of Internet use, categorized by online learning, socialization, and entertainment, would augment the depth of the study. Consequently, drawing on data from the China Family Panel Studies (CFPS) in 2016 and 2020, this study employs benchmark regression estimation and the propensity score matching (PSM) method to discern the net effect of Internet use on the cognitive ability of rural left-behind children. This empirical investigation aims to contribute substantively to the understanding of cognitive ability enhancement among rural left-behind children.

## Literature review

Cognitive ability is the human capacity to extract, store, and utilize information from the objective world, underscored by a comprehensive understanding of the laws governing the movement, change, and developmental trajectories of phenomena. This primarily encompasses abstract thinking, logical deduction, memory, and other cognitive skills (8). New human capital theory regards the development and formation of cognitive ability as a dynamic process and posits that cognitive ability has high plasticity in the stage of childhood (9). The impact of cognitive ability extends uniformly across all facets and stages of an individual’s development.

Children’s cognitive ability was influenced by various factors, including parental companionship, educational expectations, and family migration. Extensive research indicated that prolonged parental absence in rural Chinese families adversely affected children’s cognitive development and academic performance (10). Wu et al. (11) delved deeper into the distinctions in cognitive ability among children ‘left behind by father/mother/both parents’ separately. The findings underscored the significant role mothers played in children’s development, with their absence exhibiting a more pronounced negative impact on children. Concurrently, the disparity in educational resources and its implications for the cognitive development of rural left-behind children could not be overlooked. As the Internet became increasingly pervasive in people’s lives, a growing body of research commenced to scrutinize the influence of the Internet on children’s cognitive ability.

There was no conclusive consensus regarding the impact of Internet usage on the cognitive ability of children. One perspective posited that Internet utilization fostered the enhancement of children’s cognitive capacities by offering convenient and cost-free communication channels that facilitated information exchange. Orben and Przybylski (12) noted that a moderate level of Internet use, such as 1–2 h a day, was associated with slightly higher levels of psychological functioning. Buchanan argues that computers and the Internet are some of the best technologies for cognitive enhancement because they open the possibility for instantly accessing information, anytime and anywhere (13). Cui (14) asserted that a substantial portion of youths’ social cognition emanated from online interactions. Some argue that transferring memories to the Internet can be advantageous as it facilitates the more efficient allocation of cognitive resources. Therefore, employing Internet-based devices can foster more creative thinking and problem-solving abilities (15, 16). Sina et al. (17) found that extensive smartphone/internet exposure was associated with higher impulsivity and cognitive inflexibility scores, especially in girls.

Another viewpoint suggested that the mixed messages conveyed through the Internet might have adversely affected the cognitive development of children who lacked the ability to discriminate. Fitz and Reiner (6) and other researchers noted that Internet use also reduced cognitive ability. Hadlington (18) analyzed the issue from a biological perspective, suggesting that Internet use might have caused impaired attention. Moreover, the Internet had the potential to impede the cognitive development of adolescents by inducing Internet addiction and subsequent loss of focus. Zhou and Ding (19) found that Internet use time is not linked to cognitive ability among Chinese junior middle school students.

## Materials and methods

### Data processing

CFPS devised two distinct sets of cognitive measures, employing an alternating annual schedule, thereby accumulating a total of four sets of inquiries. One set of questionnaires including both a word test and a math test (used in 2010, 2014, and 2018). Conversely, an additional set of surveys comprised evaluations of memory and sequential aptitude, administered during 2012 and 2016. To maintain data consistency, our investigation exclusively relied on the datasets derived from Chinese Family Panel Studies 2016 and 2020 (CFPS2016&2020). The study was conducted by the China Social Science Survey Center of Peking University and reflected the social and economic problems in the country by tracking and collecting data at three levels: individual, family, and community.

The individual database within CFPS comprised highly detailed information, encompassing variables like mathematical and verbal scores obtained from cognitive ability assessments at the individual level. Utilizing personal IDs enabled the integration of the individual database with the child proxy database, facilitating the acquisition of comprehensive personal information; Employing the household sample code facilitated the connection between the individual database and the family economic database, allowing access to data on household income and related details; By utilizing the father and mother sample codes, the individual database could be correlated with parental information, thereby extracting details about the parents of the surveyed respondents. This procedural approach enabled the aggregation of both individual and familial data, laying the foundation for subsequent research and analysis.

In this investigation, the term “rural left-behind children” refers to individuals aged 17 years or younger with rural household registration, whose parents are working outside and are unable to live with their parents under normal circumstances (20). Consequently, the study sample should meet the following three criteria: (1) Aged 17 years or younger. (2) Rural left-behind children. (3) Either or both of the parents had been employed for 6 months or longer. Participants were asked, “How long did you live with your father or mother in the past 12 months?” for result assessment. Excluding samples with incomplete interviews or survey results, a valid sample of 1,669 rural left-behind children, comprising 833 boys and 836 girls, was ultimately obtained.

### Measurements

#### Dependent variable

The dependent variable in this study is “child’s cognitive ability” of rural left-behind children. Cognitive ability is defined as the brain’s capacity to process information. This study chose the scores of questions reflecting number series ability and memory ability in the CFPS questionnaire to measure cognitive ability. The total cognitive ability score, serving as a proxy variable for children’s cognitive ability, is obtained by first standardizing the ‘number series tests’ and ‘memory test’ within the CFPS modules for the years 2016 and 2020, and then summing their scores.

#### Independent variable

The independent variable in this context is the decision whether to use or not use the Internet. Drawing from the study conducted by Wang and Liu (21), the CFPS2016 and CFPS2020 questionnaires posed two inquiries: “Do you use mobile devices, such as a mobile phone or tablet PC, for Internet access?” and “Do you use a computer to access the Internet?” A positive response to either question results in the assignment of a value of 1 to the variable, while a negative response leads to the assignment of a value of 0.

#### Control variable

The developmental juncture spanning ages 10–15 demarcates a pivotal epoch in the cognitive maturation of children, wherein their comprehensive advancement is notably molded by individual determinants, scholastic tutelage, familial milieu, and assorted influential factors. Consequently, the study incorporates control variables sourced from the tripartite dimensions elucidated in Table 1, discerned from extant scholarly discourse and gleaned data originating from CFPS 2016 and 2020.

**Table 1:** Descriptive statistics of variables.

Characteristic	Total (*N* = 1,669)	Internet use		*p*-value
		No (*N* = 841)	Yes (*N* = 828)	
Cognitive ability	12.1 ± 2.9	11.4 ± 2.9	12.8 ± 2.8	<0.001
**Gender**				
Female	836 (50.1%)	455 (54.1%)	381 (46%)	0.001
Male	833 (49.9%)	386 (45.9%)	447 (54%)	
Sleep duration	8.9 ± 1.1	9.1 ± 1.1	8.6 ± 1.0	<0.001
**Reading**				
No	426 (25.5%)	244 (29%)	182 (22%)	0.001
Yes	1,243 (74.5%)	597 (71%)	646 (78%)	
Weekly television viewing time	7.7 ± 9.5	7.8 ± 8.9	7.6 ± 10.0	0.672
**Demonstration school**				
No	1,174 (70.3%)	621 (73.8%)	553 (66.8%)	0.002
Yes	495 (29.7%)	220 (26.2%)	275 (33.2%)	
Specialized class				<0.001
Not in a specialized class	310 (18.6%)	162 (19.3%)	148 (17.9%)	
No distinction	1,055 (63.2%)	556 (66.1%)	499 (60.3%)	
In a specialized class	304 (18.2%)	123 (14.6%)	181 (21.9%)	
Student cadre				0.001
No	1,191 (71.4%)	630 (74.9%)	561 (67.8%)	
Yes	478 (28.6%)	211 (25.1%)	267 (32.2%)	
Total amount of cash and deposits (logarithmic scale)	6.3 ± 4.5	5.5 ± 4.7	7.0 ± 4.2	<0.001
Education expenditures in the past 12 months (logarithmic scale)	7.1 ± 2.5	6.9 ± 2.4	7.4 ± 2.5	<0.001
Care about the child’s education				0.003
Very unconcerned	12 (0.7%)	10 (1.2%)	2 (0.2%)	
Unconcerned	219 (13.1%)	96 (11.4%)	123 (14.9%)	
Neutral	614 (36.8%)	338 (40.2%)	276 (33.3%)	
Concerned	694 (41.6%)	334 (39.7%)	360 (43.5%)	
Very concerned	130 (7.8%)	63 (7.5%)	67 (8.1%)	
**Residential area**				
East areas	282 (16.9%)	97 (11.5%)	185 (22.3%)	<0.001
Central areas	476 (28.5%)	194 (23.1%)	282 (34.1%)	<0.001
West areas	911 (54.6%)	550 (65.4%)	361 (43.6%)	<0.001

Continuous variables are presented as mean ± standard deviation, and categorical variables are displayed as percentage. *P*-value were obtained by *t*-test.

### Statistical analysis

Descriptive statistics were undertaken to scrutinize the characteristics of the participants. This entailed the computation of means and standard deviations for continuous variables, along with an examination of the distributions pertaining to categorical variables. The comprehensive descriptive statistical analysis of all variables is presented in Table 1. Subsequently, an Ordinary Least Squares (OLS) regression model was initially employed to explore the potential influence of Internet use on the cognitive ability of rural left-behind children. Concurrently, to mitigate potential endogeneity concerns, this study adopted propensity score matching (PSM) as a methodological approach. The statistical analyses were executed using R software version 4.1.0.

## Results

### Descriptive analysis

Table 1 presented a comparison between individuals who used Internet and those who did not use Internet based on various covariates. Among the 1,669 participants, 828 participants used Internet. The cognitive ability scores ranged from 2 to 19.55, with a mean of 11.4 and a variance of 2.9.

Of all the respondents, there were 836 (50.1%) females and 833 (49.9%) males. The majority of participants were found to be avid readers, constituting 74.5% of the surveyed group. Additionally, a substantial proportion of participants, 70.3%, were not affiliated with demonstration schools, while 71.4% did not identify as part of the student cadre. More than half of the participants lived in west areas (54.6%).

### Benchmark regression

The VIF test indicates that the mean VIF of the independent variables is 1.108, and the maximum VIF is 1.188, both well below the threshold of 10. Hence, no multicollinearity issues exist among the independent variables.

In this study, an Ordinary Least Squares (OLS) regression model is employed for research analysis. Model 1, Model 2, Model 3, and Model 4 all feature cognitive ability as the dependent variable, with Internet use serving as the independent variable. Model 1 concentrates on a sample consisting of left-behind children, while Model 2 is centered on non-left-behind children. Model 3 is based on data from the CFPS2016, and Model 4 utilizes data from the CFPS2020.

In Table 2, Model 1, Model 2, Model 3, and Model 4 collectively demonstrate a noteworthy positive correlation between Internet usage and cognitive ability. Based on Models 3 and 4, Internet use demonstrates a statistically significant and positive association with the augmentation of cognitive ability among rural left-behind children in 2020, in contrast to their counterparts in 2016.

**Table 2:** Results of regression analysis.

Variables	Model 1	Model 2	Model 3	Model 4
	Left-behind children	Non-left-behind children	CFPS 2016	CFPS 2020
Internet use	0.898^***^ (0.140)	0.959^***^ (0.167)	0.501^***^ (0.185)	2.361^***^ (0.206)
Gender	0.420^***^ (0.134)	0.613^***^ (0.152)	0.422^**^ (0.175)	0.506^***^ (0.185)
Sleep duration	−0.235^***^ (0.063)	−0.355^***^ (0.076)	−0.202^**^ (0.084)	0.217^**^ (0.090)
Reading	0.865^***^ (0.156)	0.838^***^ (0.174)	0.541^***^ (0.199)	1.664^***^ (0.230)
Weekly television viewing time	−0.004 (0.007)	−0.020^**^ (0.008)	−0.004 (0.009)	−0.017^*^ (0.010)
Demonstration school	0.205 (0.152)	−0.566^***^ (0.189)	0.180 (0.212)	0.625^***^ (0.198)
Specialized class	0.244^**^ (0.115)	0.350^**^ (0.143)	0.074 (0.157)	0.511^***^ (0.151)
Student cadre	0.249^*^ (0.150)	0.624^***^ (0.170)	0.092 (0.198)	0.528^***^ (0.203)
Total amount of cash and deposits (logarithmic scale)	0.056^***^ (0.015)	0.013 (0.016)	0.049^**^ (0.019)	0.096^***^ (0.022)
Education expenditures in the past 12 months (logarithmic scale)	−0.002 (0.027)	0.081^***^ (0.031)	0.113^**^ (0.049)	−0.220^***^ (0.031)
Care about the child’s education	0.820^***^ (0.080)	0.300^***^ (0.087)	0.573^***^ (0.102)	1.260^***^ (0.114)
East areas	0.469^**^ (0.189)	1.082^***^ (0.187)	0.579^**^ (0.237)	−0.182 (0.315)
Central areas	0.872^***^ (0.156)	0.561^***^ (0.199)	0.859^***^ (0.212)	0.488^**^ (0.215)
N	1,669	1,377	1,038	631
R^2^	0.194	0.188	0.117	0.486

^*^p < 0.05; ^**^p < 0.01; ^***^p < 0.001.

In Model 1, the control variables indicate that cognitive ability of rural left-behind children show a positive association with being male, maintaining regular reading habits, enrollment in specialized classes, holding a student cadre position, having higher total family savings, and residing in central or eastern areas. Conversely, there is a significant negative correlation between sleep duration and cognitive ability, indicating that individuals with longer sleep durations tend to exhibit lower cognitive functioning. No statistically significant relationship is observed between weekly television viewing time, attendance at a demonstration school, education expenditures in the past 12 months, and cognitive ability.

### Endogenous treatment: instrumental variables approach

Endogeneity concerns may arise in examining the relationship between Internet use and the cognitive ability of rural left-behind children. Primarily, certain variables with inherent difficulties in quantification may exert an impact on the cognitive ability of these children, leading to omitted variable bias and potential distortion in the regression outcomes. Additionally, there exists the possibility of reciprocal causation, where the cognitive ability of rural left-behind children may influence their Internet use, introducing a reverse causality issue and bias into the model estimates.

Addressing these challenges, an instrumental variable approach becomes imperative to mitigate endogeneity and derive the net effect of Internet use on the cognitive ability of rural left-behind children. Building upon the research framework established by Zhang and Tan (22), this study employs the “average score of importance of information channels “as an instrumental variable for Internet use. The validity of instrumental variables is contingent upon two critical conditions. Firstly, relevance is crucial, indicating a close association between the Internet development status and the Internet use of rural left-behind children. Secondly, exogeneity is essential, signifying that the average score of importance of information channels is unlikely to directly impact the cognitive ability of rural left-behind children. This ensures the fulfillment of exogeneity, a fundamental requirement for the validity of instrumental variables.

To address the endogeneity concern within the regression model, the 2SLS method is conventionally employed for scrutinizing both the endogeneity of the model and the efficacy of instrumental variables (23). This study also employs this method to assess the instrumental variables, presuming the presence of endogeneity in the model. The F-statistic for the weak instrument test is 51.57, significantly surpassing 10, signifying the effectiveness of the instrumental variable “average score of importance of information channels” (Table 3).

**Table 3:** Endogeneity analysis results (2SLS model).

Variables	First-stage regression results	Second-stage regression results
Instrumental variables (average score of importance of information channels)	0.150^***^ (0.008)	
Internet use		1.090^***^ (0.338)
Control variables	Control	Control
*N*	1,669	1,669
$R^{2}$	0.288	0.192
First-stage *F*-value	51.57	

^***^p < 0.001.

According to Table 3, the coefficient of the first-stage instrumental variable “average score of importance of information channels” is 0.150, which is significant at the 1% level, and the coefficient of the endogenous variable Internet use in the second-stage regression is 1.090. In summary, the regression results based on instrumental variables are deemed robust and effective.

### Robustness analysis

To address the inherent issue of selectivity bias in social science research, this study employs the propensity score matching (PSM) method. PSM initially categorizes Internet use, seeking to minimize significant differences in characteristic variables between the matched treatment and control groups through simulated random grouping. The method divides the sample into treatment and control groups, approximating a natural experiment by re-matching and re-sampling the sample data, thereby bolstering the scientific validity of the results. The control group, composed of rural left-behind children who do not use the Internet, is matched with the treatment group, consisting of those who use the Internet, using kernel matching.

In general, a smaller standard deviation after matching is indicative of better results. If the absolute value of the standard deviation is less than 20%, the propensity score matching is considered more reliable; conversely, a higher absolute value suggests a less effective matching (24). As illustrated in Table 4, the bias of the variables significantly decreased after matching compared to before matching, with the standard deviation mostly controlled below 7%. The means of the treatment and control groups were closer after matching, and no significant difference between the two groups was observed, indicating that the sample selectivity bias was largely eliminated.

**Table 4:** Robustness tests.

Variables		Average value		Mean Difference	Difference *T*-test	
		Treatment group	Control group		*T*-value	*P*-value
Gender	Before matching	0.540	0.459	16.200	3.31	0.001
	After matching	0.538	0.528	1.9	0.38	0.703
Sleep duration	Before matching	8.611	9.101	−46.2	−9.44	0.000
	After matching	8.620	8.611	0.9	0.19	0.851
Reading	Before matching	0.780	0.710	16.2	3.30	0.001
	After matching	0.779	0.797	−4.1	−0.87	0.382
Weekly television viewing time	Before matching	7.622	7.818	−2.1	−0.42	0.672
	After matching	7.625	7.779	−1.6	−0.33	0.740
Demonstration school	Before matching	0.332	0.262	15.5	3.16	0.002
	After matching	0.329	0.301	6.0	1.20	0.229
Specialized class	Before matching	1.040	0.954	14.2	2.91	0.004
	After matching	1.036	0.995	6.8	1.38	0.168
Student cadre	Before matching	0.322	0.251	15.9	3.24	0.001
	After matching	0.319	0.314	1.2	0.24	0.807
Total amount of cash and deposits (logarithmic scale)	Before matching	7.038	5.543	33.8	6.90	0.000
	After matching	7.031	7.034	−0.1	−0.01	0.989
Education expenditures in the past 12 months (logarithmic scale)	Before matching	7.365	6.880	19.6	4.01	0.000
	After matching	7.358	7.394	−1.5	−0.32	0.748
Care about the child’s education	Before matching	3.443	3.409	4.1	0.83	0.406
	After matching	3.439	3.414	3.0	0.61	0.542

The results in the table are obtained using the kernel matching method. Individuals using the internet constitute the treatment group, while individuals not using the internet serve as the control group.

To make the test results more robust, this paper uses three methods, kernel matching, radius matching, and K-nearest neighbor matching, to estimate the average treatment effect (ATT), which is the average net effect of the effect of Internet use on the cognitive ability of rural left-behind children. The results of the mean treatment effects for the full sample propensity score matching are shown in Table 5. The matched results show that the mean treatment effects obtained by the three methods are 0.155, 0.167, and 0.207, respectively, with positive and significant coefficients, and the significance and sign of the regression coefficients obtained by the three matching methods are consistent, indicating that the influence of Internet use on the cognitive ability of rural left-behind children has a consistent and stable effect.

**Table 5:** Average processing effects of Internet use on the cognitive ability of rural left-behind children.

Matching method	Treatment group (1)	Control group (2)	ATT differential (1)–(2)	Standard deviation	T-value
Before matching	12.830	11.411	1.419	0.139	10.15^***^
After matching					
Kernel matching	12.812	11.756	1.055	0.155	6.80^***^
Radius matching	12.814	11.713	1.100	0.167	6.56^***^
K-nearest neighbor matching	12.812	11.941	0.871	0.207	4.21^***^

^***^p < 0.001. Individuals using the internet constitute the treatment group, while individuals not using the internet serve as the control group.

### Heterogeneity analysis

Heterogeneity prevails among rural left-behind children with distinct individual characteristics. This study assesses gender, age, left-behind type, demonstration school, and care about the child’s education to examine this heterogeneity, and the regression results are presented in Table 6.

**Table 6:** Heterogeneity test of the effect of Internet use on the cognitive ability of rural left-behind children.

Variables	Gender		Age		Types of left-behind children		
	Girls	Boys	10–12 years old	13–15 years old	Left behind children with absent father	Left behind children with absent mother	Left behind children with both parents
Internet use	0.874^***^ (0.203)	0.791^***^ (0.197)	1.383^***^ (0.0.201)	0.168 (0.200)	1.042^***^ (0.248)	0.666 (0.471)	0.584^**^ (0.191)
Control variables	Yes	Yes	Yes	Yes	Yes	Yes	Yes
*N*	836	833	856	813	520	150	930
$R^{2}$	0.226	0.172	0.240	0.151	0.178	0.268	0.212
	Demonstration school		Care about the child’s education				
	No	Yes	Unconcerned	Concerned			
Internet use	0.827^***^ (0.162)	1.083^***^ (0.267)	0.826^***^ (0.204)	0.915^***^ (0.193)			
Control variables	Yes	Yes	Yes	Yes			
*N*	1,174	495	845	824			
$R^{2}$	0.215	0.199	0.167	0.131			

^**^p < 0.01; ^***^p < 0.001.

As shown in Table 6, regarding gender differences, Internet use exhibited a statistically significant positive association with the enhancement of cognitive ability in girls when contrasted with boys. Concerning age heterogeneity, the cognitive ability benefits linked to Internet utilization were markedly more pronounced among rural left-behind children aged 10–12 years in comparison to those aged 13–15 years. Upon categorizing rural left-behind children into distinct groups based on the parent they are left behind by, namely “left behind by father,” “left behind by mother,” and “left behind by both parents.” Notably, the cognitive ability of left behind children with absent father demonstrates the most substantial impact resulting from Internet use. In contrast, the correlation between Internet use and cognitive ability appears to be non-significant among left behind children with absent mother.

The cognitive benefits associated with Internet use were notably more pronounced among rural left-behind children, especially when considering factors such as attendance at a demonstration school and parental concern for the child’s education.

## Discussion

Drawing on a sample of 1,669 rural left-behind children from the China Family Panel Studies (CFPS) in 2016 and 2020, an Ordinary Least Squares regression model was formulated. The ensuing results reveal that:

Firstly, there is a significant positive correlation between Internet use and cognitive ability among rural left-behind children. This finding aligns with the conclusions drawn by Chen and Gu (25) and Fang et al. (26). In addition, the results of the robustness test were consistent with the results of the baseline regression of this study. The robustness and credibility of the cognitive enhancement effect of Internet use on rural left-behind children have been confirmed. In 2020, Internet use exhibited a statistically significant and positive correlation with the improvement of cognitive ability among rural left-behind children, as opposed to their counterparts in 2016. This may be because the COVID-19 pandemic forced educational institutions worldwide to adapt to online learning. For rural left-behind children who might have had limited access to traditional educational resources, the shift to online learning could have expanded their access to educational materials and opportunities. Participating in online learning also demands the acquisition of digital literacy skills. The process of learning to utilize digital tools and navigate online platforms can play a crucial role in the development of cognitive skills, including problem-solving and critical thinking.

Secondly, the outcomes of the heterogeneity analysis reveal that among rural left-behind children, girls are more significantly influenced by Internet use compared to boys. This discrepancy can be ascribed to boys may dedicate more time to gaming, which might have a distinct impact on cognitive ability compared to the activities that girls typically engage in on the Internet, such as socializing and maintaining relationships (27). Within this age bracket, the cognitive enhancement impact of Internet usage is more conspicuous among left-behind children aged 10–12 years than among their counterparts aged 13–15 years. The observed variation can be attributed to the fact that children aged 10–12 years are typically in a crucial phase of cognitive development. During this period of cognitive development, the brains of children aged 10–12 exhibit heightened plasticity and responsiveness, making the younger age group more susceptible to the positive cognitive effects of interactive online activities, educational games, and informational resources on the Internet. On the other hand, adolescents aged 13–15 years may already have undergone certain cognitive developments. The impact of Internet use on cognitive ability during this stage might be influenced by factors such as school curriculum, peer interactions, and other extracurricular activities. Additionally, the older age group might have developed other cognitive skills or have access to a wider range of resources beyond the Internet, potentially diluting the observed impact.

This variation can be attributed to the circumstance that individuals aged 10–12 are in primary school, a pivotal phase for assimilating novel knowledge and experiences. The ample learning and entertainment resources on the Internet serve to compensate for the restricted real-life knowledge and resources, consequently elevating their cognitive ability. Furthermore, regarding the heterogeneity among types of left-behind children, the cognitive ability of left behind children with absent father exhibit the most significant impact resulting from Internet use. In contrast, the correlation between Internet use and cognitive ability seems to be non-significant among left behind children with absent mother, which is consistent with the findings from previous studies (28, 29). Furthermore, a mother’s parenting style significantly shapes a child’s development, with mothers often playing a central role in creating a healthy family environment, including providing essential guidelines and rules for children’s Internet use (30). This highlights the crucial role of mothers in the development and cognitive enhancement of rural left-behind children.

The cognitive benefits associated with Internet use were notably more pronounced among rural left-behind children, especially when considering factors such as attendance at a demonstration school and parental concern for the child’s education. The conclusion of the study is in accordance with the research findings of Borghans et al. (31) and Li et al. (32). Attending demonstration schools and exposes left-behind children to innovative teaching methods and educational technologies. The Internet is likely integrated into these approaches, offering students valuable exposure to modern learning tools and methodologies. Parental concern for their children’s education is a crucial factor in overseeing their proper use of the Internet. This concern translates into active supervision, ensuring that the Internet contributes positively to enhance children’s cognitive ability.

Drawing from the findings, this study puts forth the following recommendations:

1. Enhancing rural network infrastructure is crucial for bridging the urban–rural digital gap and fostering the cognitive development of left-behind children. The existing disparity in digital access between urban and rural areas is a pressing issue that requires immediate attention from relevant government entities. To address this challenge, prioritizing the improvement of physical Internet access in rural areas is paramount. This involves investing in the development of robust network facilities and digital infrastructure, ensuring that essential electronic equipment is readily available. By eliminating the connectivity issues prevalent in many rural regions, we can create a foundation for equal access to information and educational resources. Moreover, recognizing the limited literacy and proficiency in Internet use among rural residents is essential. Government initiatives should include comprehensive programs that not only focus on improving physical access but also on providing training and support for rural communities to enhance their digital literacy skills. This dual approach ensures that the benefits of improved infrastructure are maximized. In parallel, rural schools play a crucial role in shaping the skills and knowledge of the next generation. Introducing courses specifically designed to teach Internet use skills and media literacy will empower rural children to navigate the online world effectively. This educational emphasis aligns with the goal of harnessing the stimulating effect of the Internet on the cognitive development of left-behind children. However, the positive impact of the Internet on cognitive development must be balanced with efforts to counteract the potential negative consequences of exposure to undesirable online content. Implementing effective content filtering and parental control measures can help create a safer online environment for children. Additionally, parental guidance is pivotal in shaping children’s Internet habits. Parents should lead by example, demonstrating positive Internet usage behaviors and actively engaging with their children in the online space. Encouraging a shift from entertainment-focused Internet use to a more learning-oriented approach will contribute to the holistic development of left-behind children. In conclusion, a comprehensive strategy that combines infrastructure development, digital literacy programs, and parental guidance is essential for narrowing the urban–rural digital gap and promoting the cognitive development of rural left-behind children. This proactive approach recognizes the instrumental role of information technology and aims to harness its potential for the benefit of all communities.
2. Establish resource equilibrium in rural schools and incorporate the Internet into the curriculum. Numerous rural schools confront a considerable shortage of resources, especially in terms of teaching materials, including Internet resources, owing to insufficient funding. The infusion of high-quality resources not only empowers rural students with practical Internet skills but also opens up new learning horizons for teachers in remote areas. By prioritizing the provision of up-to-date teaching materials and incorporating technology into the curriculum, we can bridge the digital divide that often hampers the educational progress of students in rural settings. This proactive step goes beyond merely addressing resource shortages; it acts as a catalyst for innovation in teaching methodologies and prepares students for the demands of the digital era. Moreover, by investing in the professional development of teachers, we ensure that they are equipped with the necessary skills to leverage Internet resources effectively. Workshops, training programs, and mentorship initiatives can empower educators to incorporate digital tools seamlessly into their teaching practices, thereby enriching the learning experience for students. As a result, the entire educational ecosystem in rural areas stands to benefit, creating a positive ripple effect on the cognitive development and academic performance of left-behind children.
3. Prioritizing the well-being of left-behind children is paramount, especially in the context of the ongoing urbanization trend, which has led to a surge in the population of children left behind in rural areas. As more rural residents pursue employment opportunities in cities, a concerning information and emotional disconnect has emerged between urban and rural areas, impacting the physical and mental health of left-behind children. The absence of parental care has left these children vulnerable to delayed or unaddressed physical and mental health issues. To safeguard their well-being, it is imperative for the government to implement comprehensive health programs specifically tailored for left-behind children. These programs should encompass regular health check-ups, mental health support services, and educational initiatives that promote a holistic approach to their development. Recognizing the unique challenges faced by left-behind children, there is a need for targeted policies and interventions that address the root causes of their vulnerabilities. This includes initiatives to strengthen community support systems, engage local stakeholders, and create a nurturing environment that compensates for the lack of parental presence. Collaborative efforts between government agencies, non-profit organizations, and local communities can help establish a network of care and support for these children.

However, this study also has certain limitations: Firstly, the research relies on a specific sample derived from rural areas in China. Consequently, caution must be exercised in generalizing the results to other settings or extrapolating them to encompass all rural areas across China. The unique characteristics and contextual nuances of the chosen sample may restrict the broader applicability of the findings. Secondly, due to constraints on the availability of variables in secondary data measurement, there is a lack of specific analysis pertaining to the preferences and patterns of internet use. This absence may hinder a comprehensive understanding of the factors influencing internet use in the studied population.

## Conclusion

In summary, this study, based on the China Family Panel Studies (CFPS) data from 2016 and 2020, reveals a significant positive correlation between Internet use and the cognitive ability of rural left-behind children aged 10–15. Emphasizing infrastructure development, digital literacy programs, and parental guidance can effectively narrow disparities. Additionally, establishing resource equilibrium in rural schools and prioritizing the well-being of left-behind children through targeted health programs are essential steps toward holistic development.

## Data availability statement

The original contributions presented in the study are included in the article/supplementary material, further inquiries can be directed to the corresponding author.

## Author contributions

A-zG: Writing – original draft, Writing – review & editing. W-cC: Writing – original draft, Writing – review & editing.
